# Staff Numbers Matter

**DOI:** 10.1002/jgf2.486

**Published:** 2021-07-27

**Authors:** Koki Kato

**Affiliations:** ^1^ Madoka Family Clinic Ogori Japan; ^2^ Academic and Research Centre Hokkaido Centre for Family Medicine Sapporo Japan

## CONFLICT OF INTEREST

The authors have stated explicitly that there are no conflicts of interest in connection with this article.

To the Editor,

With great interest, I read the article of Tago et al.^1^ about the research practice in general medicine departments of Japanese universities. I endorse the relevance of this issue because the speciality of general medicine was just started several years ago, and the university departments are expected to contribute to research to show the value. They set the primary outcome as the number of English‐language research publications (ELRP) in three years and demonstrated that the ELRP was associated with the perceived degree of research necessity, staff numbers, collaborative research, conference presentations, and obtaining research grants. However, I have some concerns about the interpretation and presentation of the results of this study.

The number of ELRP is expected to increase where the staff numbers are high. Then, the staff numbers would be highly correlated to collaborative research, conference presentations, and obtaining research grants. Tago et al. demonstrated only univariate analysis results, but they should have demonstrated the multivariate analysis results adjusted by the staff numbers to show the productivity of ELRP. Otherwise, the primary outcome should have been set as the number of ELRP per assistant professor or above.

This study is informative for the departments of general medicine in Japan, suggesting the potential factors for increasing the number of ELRP in the departments. However, considering the above, I am concerned that the staff numbers might strongly affect the results, and it could obscure genuine successful factors for increasing ELRP. Therefore, I think we need a cautious interpretation of the results, and it would be better to inform readers of the above limitations and concerns.
